# Acoustic neuromodulation from a basic science prospective

**DOI:** 10.1186/s40349-016-0061-z

**Published:** 2016-05-20

**Authors:** Elisabetta Sassaroli, Natalia Vykhodtseva

**Affiliations:** Department of Radiology, Brigham and Women’s Hospital, Focused Ultrasound Lab, 221 Longwood Ave., Boston, MA 02115 USA

**Keywords:** Focused ultrasound, Neuromodulation, Neurostimulation, Action potential, Hodgkin–Huxley model, Electromechanical coupling, Piezoelectricity, Flexoelectricity, Cavitation, Soliton

## Abstract

We present here biophysical models to gain deeper insights into how an acoustic stimulus might influence or modulate neuronal activity. There is clear evidence that neural activity is not only associated with electrical and chemical changes but that an electro-mechanical coupling is also involved. Currently, there is no theory that unifies the electrical, chemical, and mechanical aspects of neuronal activity. Here, we discuss biophysical models and hypotheses that can explain some of the mechanical aspects associated with neuronal activity: the soliton model, the neuronal intramembrane cavitation excitation model, and the flexoelectricity hypothesis. We analyze these models and discuss their implications on stimulation and modulation of neuronal activity by ultrasound.

## Background

Neuromodulation methods, such as a deep brain stimulation, transcranial direct current stimulation, and transcranial magnetic stimulation, have attracted widespread attention due to their therapeutic effects in the treatment of neurological and psychiatric diseases [1]. However, these methods have serious limitations such as surgically implanted electrodes (deep brain stimulation), a low spatial resolution (transcranial magnetic stimulation), or genetic manipulation (opto-genetic techniques) [2]. Ultrasound can propagate through the skull bone [3, 4], focus in a small targeted volume, and interact with biological tissues through thermal and/or non-thermal mechanisms, which make it a potentially powerful neuromodulation tool.

It has been known for several decades that ultrasound can influence neuronal activity. Most of the early studies investigated effects of focused ultrasound (FUS) on the central nerve system, on peripheral nerves and spinal tract, and on sensory receptors. Here, we shortly summarize some of the important results achieved in these studies [5–16]. Fry et al. [5] were among the first researchers to study the effect of FUS on electrical activity in the brain. In 1958, this group reported that FUS applied to the lateral geniculate nucleus through the skull window caused a reversible inhibition of the electrical responses evoked in the visual cortex of cats whose eyes were stimulated by light. A partial reduction of the evoked potential occurred immediately upon FUS exposure and a complete recovery of visual functions occurred with 30 min after exposure. It was found that ultrasound effects on chemical synapses are among the earliest changes to occur, providing a possible explanation for the functional changes observed immediately following ultrasound application [6]. Similar experiments were performed by Vykhodtoseva’s group that applied FUS to the optic tract and lateral geniculate nucleus junction, also through the skull window, and recorded the visual evoked potentials in both the visual cortex and the optic tract. Extent of the suppression and degree of recovery varied depending on the ultrasound dosage used [7, 8]. In some cases, effects on visual cortex were delayed 4–5 min compared to the same effects on the optic tract. One of the possible mechanisms of the delayed effect was suggested to be spreading depolarization (SD), which is an electrochemical wave propagating through neural tissue at 2–5 mm/min causing cessation of neuronal bioelectrical activity and massive surges of extracellular potassium (>50 mM). This suggestion was tested on the rat’s brain, and the FUS possibility of inducing a negative shift of direct current potential (reflecting ionic changes in brain tissues) and to initiate SD in cortical and sub-cortical structures (cerebral cortex, caudate nucleus, thalamus, and hippocampus) were confirmed [10–12]. Effects of FUS on peripheral nerves were studied by numerous investigators since the early 1920s. The most extensive studies were performed by Lele [13], who studied the effect of FUS on the peripheral nerves of cats, monkeys, and humans. He described three different effects on the action potential (AP) depending on the exposure conditions: a reversible enhancement, progressive inhibition, and irreversible inhibition. According to Lele, all the effects induced by FUS on nerve fibers can be reproduced by the application of heat to certain regions of the nerves. Tsirulnikov with colleagues (Institute of Evolutionary Physiology and Biochemistry, Sankt-Petersburg, Russia) in collaboration with Gavrilov (Acoustical Institute, Moscow) used FUS for studying functional effects on neuroreceptor structures, in particular, for stimulation of superficial and deep-seated sensory receptors with a purpose of studying tactile, thermal, hearing, and other sensations including pain sensations (see for review [14]). They developed non-invasive methods for the diagnosis of dermatological and neurological disorders accompanied by considerable differences in sensitivity of skin and tissue sensory receptors; for diagnosis of various hearing disorders, especially in cases with complex pathology [15], and for non-invasive stimulation of the nociceptors for investigating pain in human and animals [16].

Recently, there has been a renewed interest in investigating the effects of FUS on neuronal activity [17–19], brain functions [20–27], peripheral nerves [28, 29], and on sensory receptors [30–32]. For the purpose of illustration, we briefly summarize here some of the results of these most recent investigations. Tyler’s group showed that low intensity, low-frequency (670 kHz or less) pulsed ultrasound can generate a nerve impulse and synaptic excitation transfer in hippocampal slices of the mouse brain [17]. They also reported ultrasound-induced motor activity upon transcranial insonation of the cerebral cortex of mice [20]. King et al. [21, 22] also investigated transcranial neurostimulation of the mouse somatomotor brain area. They reported neurostimulation at a frequency of about 500 kHz with an increase in efficacy by increasing the intensity of the stimulus. Younan et al. [23] investigated the pressure threshold required to induce a motor response in anesthetized rats exposed to transcranial ultrasound pulses of about 320 kHz frequency. They estimated an average acoustic pressure threshold for motor neuromodulation of 1.2 ± 0.3 MPa with MI = 2.2 ± 0.5 and *I*_SSPA_ = 17.5 ± 7.5 W/cm^2^. Yoo and collaborators have shown that low-intensity pulsed focused ultrasound, operated at a frequency of about 690 kHz, can induce a variety of non-invasive functional brain activity changes. These changes include transient modulation of the somatomotor and visual areas of the rabbit brain [24], a reduction in the epileptic activity in rats [25], a decrease in the extracellular level of the neurotransmitter GABA in rats [26], and a reduction of the time taking to anesthetized rats to recover from anesthesia [27].

Despite all experimental studies, the mechanisms through which FUS can influence neuronal activity are currently poorly understood. In this paper, we will consider more specifically the effect of ultrasound on the AP or nerve impulse. In the classical Hodgkin–Huxley (H–H) model [33], the AP is generated by the orchestrated opening and closing of the voltage-gated sodium and potassium ion channels. The H–H model explains very well the electrical aspect of the AP, but it cannot explain how a high-frequency mechanical wave such as ultrasound can influence AP. Here, we present and discuss biophysical models and hypotheses which make it possible to provide some explanation how a mechanical stimulus might influence the AP. These models include the soliton model proposed by Heimburg and Jackson [34], the flexoelectricity hypothesis proposed by Petrov [35], and the neuronal intramembrane cavitation excitation (NICE) model recently developed by Plaksin, Shoham, and Kimmel [36]. In the soliton model, the AP is suggested to be a density (sound) pulse propagating along the axon membrane as a soliton “in a manner similar to the propagation of a piezoelectric wave”. Flexoelectricity describes the fact that imposing a deformation (bending) on the membrane induces a change on the membrane electric potential; it can also work in the reverse direction, i.e., the application of a voltage, and induces a curvature in the membrane. In the flexoelectricity hypothesis, the AP is a flexoelectric wave propagating along the axon membrane. The NICE model, suggests intramembrane cavitation (ultrasound-induced nanobubbles within the two leaflets of the lipid bilayer) as a mechanism for the initiation of the AP by ultrasound.

### Action potential

The neuron is a cell specialized to pass signals to individual target cells. It has a cell body (soma) and two types of processes extending from the soma: one or more dendrites and one axon. The cell body is the metabolic center of the cell; the dendrites convey electrical signals to the cell; the axon conveys electrical signals away from the soma.

**Fig. 1:**
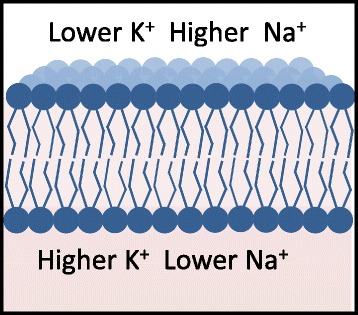
Lipid membrane bilayer. Ions are present in different concentration on the opposite sides of cell membrane

**Fig. 2:**
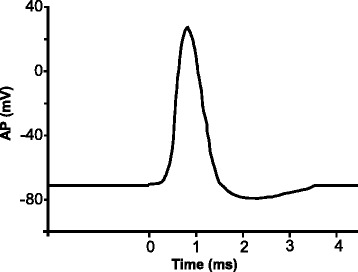
Action potential in the squid axon

In the presently accepted electrical model for the AP [37], neurons rely on changes of their membrane electrical potential as communication signals for receiving, integrating, and sending information. The cell membrane and more generally bio-membranes consist of ensembles of many different types of lipids and embedded proteins with lipids being the most numerous. Ions are present in different concentrations on the opposite side of the plasma membrane, and they determine the membrane electrical potential (Fig. 1). This potential is primary determined by the difference in concentrations of potassium (K^+^) and sodium (Na^+^) ions, with K^+^ in higher concentration inside the membrane and Na^+^ in higher concentration outside. These potentials induce the overall voltage difference between the inside and the outside the membrane, the resting potential. The resting potential of the membrane of a nerve cell is approximately −70 mV. This value refers to the inside surface of the membrane relative to the outside surface. Neurons send signals over long distance by generating and propagating APs along the axon membrane. The AP is a brief reversal of the axon membrane potential of about 100 mV. Figure 2 shows as an example the AP in the squid axon. In this figure, one may see at first a reduction of the membrane potential (depolarization): the inside membrane becomes less negative from −70 to 30 mV. Then re-polarization occurs that restores the resting potential; before the resting state is reestablished, a temporary increase of the membrane potential from −70 mV to approximately −75 mV (hyper-polarization) may occur. The duration of the AP is in the range of 1–20 ms, and AP propagates with a velocity between 0.1 and 100 m/s. This corresponds to AP lengths ranging from a few millimeters to a few centimeters.

### Action potential: Hodgkin–Huxley model

In 1952, Hodgkin and Huxley developed the theory that is the presently accepted model for the nerve pulse (Nobel Prize, 1963). They analyzed the AP in the squid axon using the voltage-clamp technique [33]. In the voltage-clamp experiments, an electrode is inserted into the axon and a voltage difference is applied between the inside and outside of axon. The H–H model postulated the existence of ionic currents flowing through specific voltage-gated sodium Na^+^ and potassium K^+^ ion channels distributed along the axon membrane. In 1976, Neher and Sakmann using the patch-clamp technique confirmed the existence of such channels [38] (Nobel Prize 1991); in 1998, the K^+^ ion channel was crystallized by MacKinnon and coworkers [39] (Nobel Prize 2003); and the mechanism for the selective transport of K^+^ and Na^+^ has been established. Ion channels are specific membrane proteins that allow only one type of ion to pass in and out the channel. For example, the K^+^ channel allows only potassium ions to pass across the membrane along its concentration gradient. Voltage-gating means that the channel can be opened only by the application of an appropriate voltage.

**Fig. 3:**
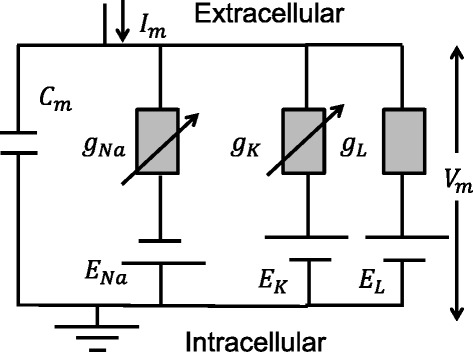
Equivalent circuit for H–H model

In the H–H model, the electrical behavior of the axon membrane is represented by an electrical circuit (Fig. 3) in which the axon membrane is a capacitor with capacitance (*C*_*m*_ ≅ 1μF/cm^2^) and the ion channels are resistors obeying Ohm’s law. When a strong depolarization is induced in the axon membrane, for example, by applying a voltage clamp step *V* that brings the membrane resting potential from −70 to 0 mV, a distinctive current density (current per cm^2^*)* flows across the membrane which is the sum of four currents: *I*_HH_ = *I*_C_ + *I*_L_ + *I*_Na_ + *I*_K_ with:

1 $$ {I}_{\mathrm{HH}}={C}_M\frac{dV}{dt}+{g}_L\left(V-{E}_L\right)+{g}_{\mathrm{Na}}\left(V-{E}_{\mathrm{Na}}\right)+{g}_K\left(V-{E}_K\right) $$

where *I*_C_ is a capacitive current, and *I*_Na_, *I*_K_, and I_L_ are ionic currents flowing through the resistors. The capacitive current (*I*_C_) results from charging the capacitor by the voltage *V*; it consists of the initial brief spike of outward current. The three ionic currents are a time-dependent inward ionic current (*I*_Na_) caused by Na^+^ ions flowing through the voltage-gated Na^+^ channels; a time-dependent outward ionic current (*I*_K_), which develops more slowly than the Na^+^ current and is produced by K^+^ ions flowing through the voltage-gated K^+^ channels; and a time independent small outward current (*I*_L_) caused by all the other ions (mainly Cl^−^ ions) flowing through the leak channels, which are open all the time. In Eq. (1), *E*_L_, *E*_Na_, and *E*_*K*_ are the equilibrium potentials. For the leak channels, *E*_L_ is the potential at which the leak current is zero. In the giant axon of squid, for example, the concentration of K^+^ outside is *c*_out_ = 20 mM and inside *c*_in_ = 400 mM; the concentration of Na^+^ is *c*_out_ = 440 mM and *c*_in_ = 50 mM [37]. From the knowledge of *c*_in_ and *c*_out_, the electric potential for a given ion type, Nernst potential, is

2 $$ {E}_{\mathrm{i}}=\frac{RT}{zF} \ln \left(\frac{c_{\mathrm{out}}}{c_{\mathrm{i}\mathrm{n}}}\right) $$

where *R* is the universal gas constant, *T* is the absolute temperature in Kelvins, *z* is the electrical charge of ion (+1 for K^+^ and Na^+^), and *F* is Faraday’s constant. For the squid axon, the Nernst potential for the K^+^ ions is *E*_k_ = − 80 mV and for Na^+^*E*_Na_ = 60 mV. The *g*_Na_, *g*_k_, and *g*_L_ are ionic conductances (inverse of resistance) that for Na^+^ and K^+^ ions are functions of time and applied voltage *V*. They are obtained by fitting the experimental data for the *I*_Na_ and *I*_K_ currents to corresponding mathematical expression in Eq. (1). The conductance for the leakage channels *g*_L_ is time independent and also obtained by fitting the experimental data to the mathematical expression.

In the H–H voltage-clamp experiments, the axon membrane experienced the same voltage *V* and there was no AP propagation. Net current different from zero appears across the membrane only during the application of the voltage clamp step for a few milliseconds before the new equilibrium condition for the membrane is reached. In cable theory, the spreading of the voltage pulse along the cylindrical membrane is described by the differential equation [33]:

3 $$ \frac{\partial^2V}{\partial {x}^2}=\frac{2{R}_i}{a}{I}_{\mathrm{HH}} $$

where *R*_i_ is the inner resistance of medium inside the neuron, *a* is the radius of the axon (cable), *I*_HH_ is the current given by Eq. (1), and *x* is the pulse propagation direction. This equation assumes that a local patch of membrane is depolarized by a strong enough depolarization potential generating the characteristic transmembrane current *I*_HH_ described above. This current acts as a stimulus for the following patch of axon membrane and the voltage pulse propagates forward. Taking into account that the pulse propagates with constant speed *v,* the wave equation: $\frac{{\mathit{\partial}}^2V}{\mathit{\partial}{t}^2}={v}^2\frac{{\mathit{\partial}}^2V}{\mathit{\partial}{x}^2}$ can be used in Eq. (3). Combining Eq. (1), Eq. (3), and the wave equation, the differential equation for the AP in the H–H model is

4 $$ \frac{a}{2{R}_i{v}^2}\frac{{\mathit{\partial}}^2V}{\mathit{\partial}{t}^2}={C}_{\mathrm{M}}\frac{dV}{dt}+{g}_{\mathrm{L}}\left(V-{E}_{\mathrm{L}}\right)+{g}_{\mathrm{Na}}\left(V-{E}_{\mathrm{Na}}\right)+{g}_{\mathrm{K}}\left(V-{E}_{\mathrm{K}}\right) $$

The solution of this equation reproduces very well the experimental data for the generation, propagation, and shape of the AP (Fig. 1). Thus, in the Hodgkin and Huxley model, AP is a purely electrical phenomenon based on conductors (ion channels and cytosol of the axon) and on the capacitor, which is the lipid membrane.

Most nerves are not as simple as the squid axon; they contain more than two voltage-gated ion channels. In particular, the mammalian central neurons typically show dozen different types of ion channels. These channels allow encoding information by generating action potentials with a wide range of shapes, frequencies, and patterns [40]. Modifications of the H–H model including these multiple ion channels have been developed (see for example [41–45]).

### Non-electric aspects of action potential

There are, however, a number of thermodynamic findings on nerves that are not contained in the classical H–H theory. Particularly remarkable is the finding of reversible heat changes as well as thickness and length changes in the axon membrane during AP propagation. It has been observed by a number of investigators that the dimensions of the nerve change in phase with voltage changes and that the nerve exerts a force normal to the membrane surface. In a series of publications [46–48], Tasaki and collaborators have shown that the AP is accompanied by an upward displacement of the nerve surface of about 1 nm, a kind of swelling with the peak coinciding with the peak of the action potential. Simultaneously with this displacement, a longitudinal shortening of the nerve was observed. The onset of this longitudinal shortening reflected the time required for the AP to propagate between the stimulating cathode and the observation point. Tasaki and his group observed this swelling accompanying the AP in all the excitable cells and tissues that they tested including invertebrate and vertebrate nerve fibers [49]. Although some swelling is always associated with the exchange of Na^+^ and K^+^, the upward displacement during the AP was about two orders of magnitude larger than the one measured for Na^+^ and K^+^ exchange. More recently, the advanced method of optical coherence tomography confirmed membrane displacement in the nanometer range with sub-nanometer accuracy [50, 51]. The time evolution of the optical signal was roughly synchronous with the action potential. In 2015, Alfredo Gonzalez-Perez and colleagues [52] using atomic force microscopy (AFM) found the vertical displacement to be between 2 and 12 Å, lasting between approximately 2 and 4 ms, during AP propagation in the giant axons of the lobster.

Particularly striking is also the finding of reversible heat changes in the membrane during the AP. A number of authors have shown that, within experimental errors, heat released during the initial phase of AP is reabsorbed in the final phase of AP [53–57]. During the nerve pulse, no heat (or very little) is dissipated, so that the entropy of the membrane is basically conserved [58, 59]. This reversible heat production in the microkelvin temperature range and the millisecond time scale suggest that the process is adiabatic in contrast to the dissipative nature of AP postulated by Hodgkin [60] who compared AP with “burning of a fuse of gunpowder”.

### Action potential: soliton model

Heimburg and Jackson [34] have proposed that the AP is “a propagating density pulse (soliton), and therefore an electromechanical rather than a purely electrical phenomenon”. The soliton model is based on the thermodynamics and phase behavior of the lipids in the cell membrane. A soliton or solitary pulse is known to be a localized pulse propagating without attenuation and without change of shape. In mathematical physics, two conditions are necessary for the existence of solitons: the pulse speed should be frequency dependent and a non-linear function of the pulse amplitude. Within lipid phase transition, both conditions for existence of solitons: dispersion and non-linearity of the speed of sound are present and a soliton can propagate along the axon membrane.

#### Lipid phase transitions

**Fig. 4:**
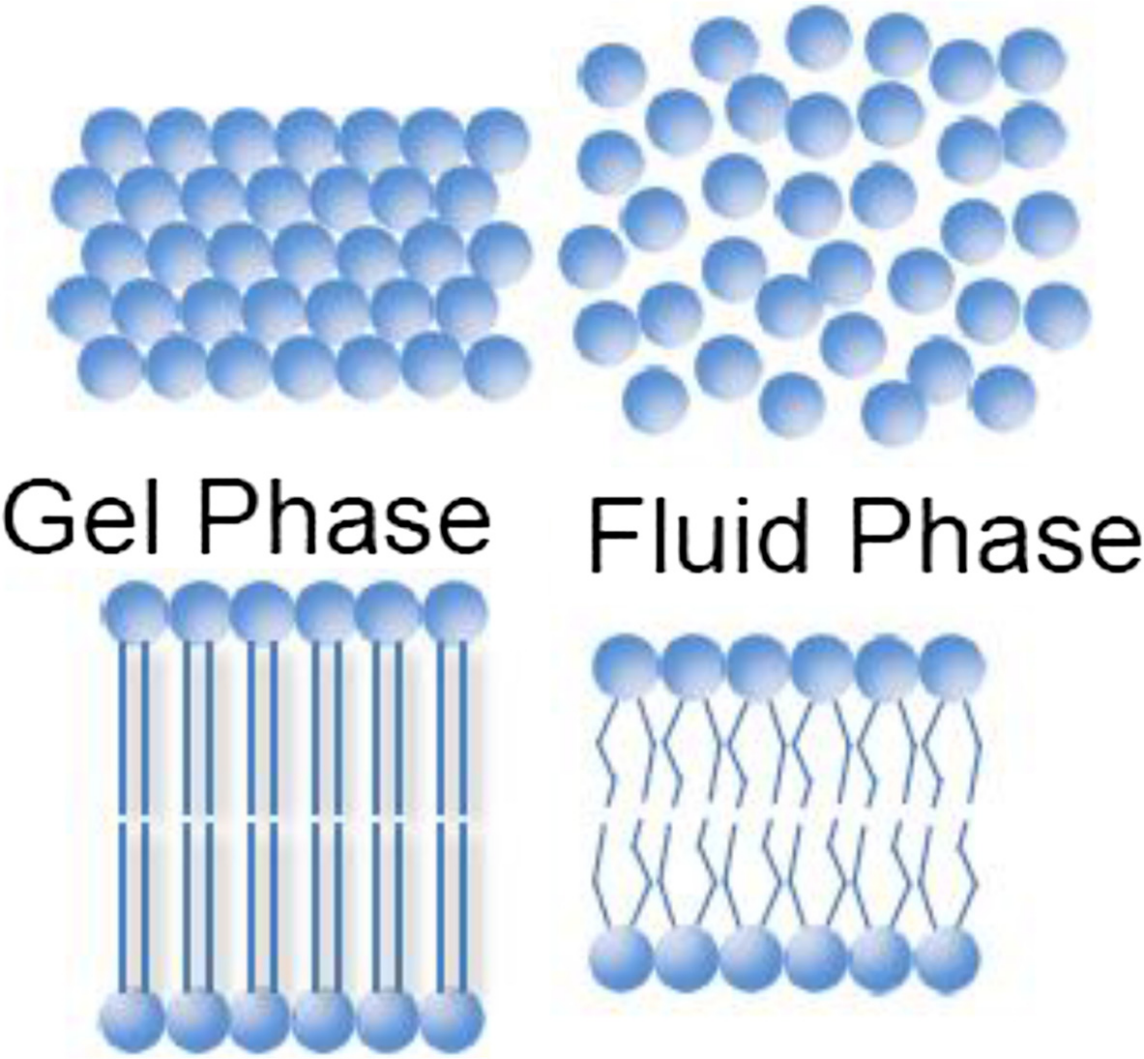
Gel and fluid phases. In the gel phase, the area and the volume of the lipid membrane are minimal, while its thickness is maximal

In the currently accepted model of biological membranes, the lipids are assumed to be in the fluid state with some domains in the gel phase [61]. Lipids display reversible phase transitions from the liquid to the gel phase (Fig. 4) and vice versa. While changes of all the intensive thermodynamic variables: temperature, voltage, pressure, lateral (shear) pressure (a stress applied transversely to the direction normal to lipid bilayer), and chemical potentials (such as pH, calcium concentration) can influence phase transitions (see [61] for a review), phase transitions induced by temperature have been the most studied. Phase transitions can be measured using calorimetry, which measures the heat capacity: *c*_p_ = (dQ/dT)_p_ of the sample (heat (*dQ*) uptaking per temperature increment (*dT*) under constant pressure). At phase transition, the heat capacity displays a maximum, due to the energy required to change the molecular arrangement from the gel to the fluid phase. Phase transitions have been investigated on biological membranes such as *Escherichia coli*, *Bacillus subtilis* membranes, and bovine lung surfactant [61]. The lipids in these membranes display phase transitions close to the physiological range of temperatures. The bovine lung surfactant membrane displays a broad phase transition profile with a maximum at about *T*_m_ = 27 °C. Gel and fluid phases are associated with different values of area, thickness, and volume of the membrane. In the gel phase, the specific area (area/mass) and specific volume (volume/mass) of membrane are minimal, while its thickness and area density, which is the inverse of the specific area, are maximal. For instance, the artificial lipid membrane dipalmitoylphosphatidylcholine (DPPC) exists in the gel phase at room temperature and shows a reversible phase transition at temperature of 41.3 °C. It has a specific volume of about 0.95 cm^3^/g in the gel phase, and about 1.00 cm^3^/g in the fluid phase corresponding to a relative specific volume decrease of about $\frac{V_{\mathrm{fluid}}-{V}_{\mathrm{gel}}}{V_{\mathrm{fluid}}}=5\%$ [62]. In addition, the DPPC lipid membrane specific area is 1.90 × 10^6^ cm^2^/g in the gel phase and 2.52 × 10^6^ cm^2^/g in the fluid phase corresponding to a relative specific area decrease of $\frac{A_{\mathrm{fluid}}-{A}_{\mathrm{gel}}}{A_{\mathrm{fluid}}}=25\%$ and relative increase in thickness of $\frac{D_{\mathrm{fluid}}-{D}_{\mathrm{gel}}}{D_{\mathrm{fluid}}}=-16\%$. These values vary for different lipids but provide the order of magnitude of the effect.

#### Soliton model equation

In the soliton model, the wave equation is expressed in terms of the area density difference: *Δρ*_*A*_ = *ρ*_*A*_ − *ρ*_*A*,fluid_ as

5 $$ \frac{{\mathit{\partial}}^2\varDelta {\rho}_A}{\mathit{\partial}{t}^2}=\frac{\mathit{\partial}}{\mathit{\partial}x}\left({v}^2\frac{\mathit{\partial}\varDelta {\rho}_A}{\mathit{\partial}x}\right)-h\frac{{\mathit{\partial}}^4\varDelta {\rho}_A}{\mathit{\partial}{x}^4} $$

where *ρ*_*A*,fluid_ is the area density in the fluid phase slightly above phase transition, *x* is the direction of propagation of the wave, and *t* is time*.* The last term in the right-hand side of Eq. (5) is the term responsible for dispersion. The speed of sound *v* is a non-linear function of the area density difference:

6 $$ {v}^2={c}_0^2+a\varDelta {\rho}_A+b{\left(\varDelta {\rho}_A\right)}^2 $$

with *c*_0_ speed of sound in the fluid phase just above phase transition and *a* and *b* membrane dependent constants. Equations (5) and (6) are assumed to be valid only close to the phase transition and within the phase transition range.

The solution of the wave equation Eq. (5) which represents a soliton pulse is [63]:

7 $$ \frac{\varDelta {\rho}_A}{\rho_{A,\mathrm{fluid}}}=\frac{a}{b}\frac{1-\frac{v^2-{v}_{\min}^2}{c_0^2-{v}_{\min}^2}}{1+\sqrt{\frac{v^2-{v}_{\min}^2}{c_0^2-{v}_{\min}^2}} \cosh \left(\frac{c_0}{\sqrt{h}}z\sqrt{1-\frac{v^2}{c_0^2}}\right)} $$

with *z* = *x* − *vt*. The pulse propagates on the membrane along the axial direction *x* with speed *v*. The value of the dispersion parameter *h* sets the width of the pulse. The solution of the soliton equation (Eq. (5)) exists only when the speed *v* is between *c*_0_ and the minimum speed given by:

8 $$ {v}_{\min }=\sqrt{c_o^2-\frac{a^2}{6b}} $$

The minimum speed corresponds to the maximum possible area density change expressed by

9 $$ \varDelta {\rho}_{A, \max }=\frac{\left|a\right|}{b} $$

**Fig. 5:**
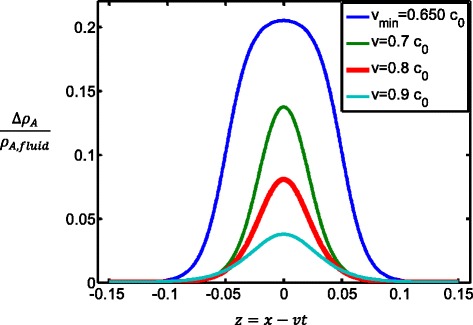
Soliton profiles along the DPPC lipid membrane

For a DPPC lipid membrane at the temperature of 45 °C (just above the phase transition), the parameters in all the above equations have been estimated as ${\rho}_{A,\mathrm{fluid}}=4.035\times {10}^{-3}\frac{g}{{\mathrm{m}}^2}$, ${c}_0=176.6\frac{m}{s}$, $a=-16.6\frac{c_0^2}{\rho_{A,\mathrm{fluid}}}$, and $b=79.5\frac{c_0^2}{{\left({\rho}_{A,\mathrm{fluid}}\right)}^2}$ [34]. By substituting these values into Eq. (8), the minimum speed is ${v}_{\min }=115\frac{m}{s},$ which is very close to the speed of the AP in myelinated nerves. Soliton profiles for the DPPC lipid membrane for velocities between *v*_min_ and 0.9*c*_0_ are shown in Fig. 5. The largest profile corresponds to lowest velocity *v*_min_; the parameter *h* is equal to $h=2\frac{m^4}{s^2}$ generating a pulse of a few centimeters width, which can be found in some nerves. The maximum change of the area density, given by Eq. (9) divided by *ρ*_*A*,fluid_ is $\frac{\varDelta {\rho}_{A, \max }}{\rho_{A,\mathrm{fluid}}}=0.21$. The area density is the inverse of the specific area. From the values of the specific area for the fluid and gel phases of the DPPC membraned given earlier, one can calculate the ratio between the area densities in the gel and fluid phases for the DPPC membrane as $\frac{\rho_{A,\mathrm{gel}-}{\rho}_{A,\mathrm{fluid}}}{\rho_{A,\mathrm{fluid}}}=0.32$. Thus, at maximum amplitude, the acoustic soliton exerts a lateral pressure on the lipid membrane that induces 66 % of the increase in area density occurring during the DPPC phase transition from fluid to gel. The corresponding decrease of the relative specific area during the soliton propagation is therefore 16.5 %, and the decrease of the relative specific volume is 3.3 %. For the DPPC membrane, the change in thickness between the two phases is about 7.4 Å [34] corresponding in our example to a maximum increase in the membrane thickness of about 4.9 Å. This results in the total change in the thickness of a membrane cylinder of 9.8 Å. In addition, the relative shortening of a cylindrical DPPC membrane of volume *V* = πD^2^L (D thickness L length) can be estimated from the knowledge of the relative increase in membrane thickness of 16 % and relative decrease of specific volume of 5 % from the fluid to the gel phase. The relative shortening DL/L fluid is about 29 % between the two phases, 66 % of this value corresponds to a shortening of the axon cylindrical membrane of 19 %.

#### Action potential

In the soliton model, the AP is a reversible density pulse propagating along the axon membrane. This can explain the non-electric aspects associated to AP propagation such as changes in axon membrane thickness and length, as well as heat release during membrane depolarization and heat reabsorption during repolarization. However, the AP is known to be a propagating voltage pulse with a net voltage change of about 100 mV. Heimburg and Jackson [64] have suggested that this voltage change is proportional to the change of membrane area density, and it is a consequence of the piezoelectric property of the cell membrane. Therefore, the soliton pulse propagates “in manner similar to a piezoelectric wave”.

#### Piezoelectricity in soliton model

Piezoelectric effect is the appearance of an electrical potential (a voltage) across the sides of a piezoelectric material subjected to mechanical stress. Biological membranes have properties between those of conventional liquids and those of crystals (when in gel phase) and, therefore, can be considered as liquid crystals. Piezoelectricity and flexoelectricity are thought to arise from both the large electric dipole moments of the lipid molecules and from the asymmetry in the distribution of negatively charged lipids (typically about 10 %) found primarily in the inner leaflet of the bilayer [65, 66]. Proteins, which can carry both positive and negative charges, are also asymmetrically distributed and are also thought to provide a large contribution to piezoelectricity and flexoelectricity.

If a biomembrane is piezoelectric, polarization charges are induced on the opposite sides of the membrane by compressing/stretching/shearing the membrane. In membranes, there is a coupling between changes of thickness and area: a change *dD* in thickness is associated with a change in membrane area *dA*; the membrane potential which results from the piezoelectric effect is [67, 68]

10 $$ {V}_{\mathrm{piezo}}={f}_{\mathrm{piezo}}dA $$

where *f*_piezo_ is the piezoelectric coefficient, which is currently not known. The charge on the capacitor (membrane) induced by *V*_piezo_ is *q* = *C*_M_*V*_piezo_. In this equation, it is assumed that the membrane capacitance *C*_M_ does not change during area changes. The membrane capacitance can be expressed as ${C}_{\mathrm{M}}={\varepsilon}_0{\varepsilon}_{\mathrm{M}}\frac{A}{D}$ with *ε*_0_ vacuum dielectric constant of membrane, *A* membrane area and *D* (about 5 nm) its thickness. In a more general expression that takes into account also changes in *C*_M_ as a function of area changes, the charge is: $q={C}_{\mathrm{M}}{V}_{\mathrm{piezo}}+\left(V+{V}_{\mathrm{piezo}}\right)\frac{\partial {C}_M}{\partial A}dA$ with *V* applied voltage. In the inverse piezoelectric effect, the application of a voltage *V* induces a change in membrane area as [67, 68]:

11 $$ dA={f}_{\mathrm{piezo}}V $$

The role of piezoelectricity in the generation and propagation of the AP should be investigated. However, an order of magnitude estimate of the voltage change associated with area changes during AP propagation can be provided. For instance, the electrical potential induced by the charged lipids present in biological membranes can be estimated as [64]

12 $$ \varPsi =\frac{1}{\varepsilon_0\varepsilon \kappa }{\sigma}_A $$

where *ε*_0_ = 8.859 × 10^−12^ C^2^/J m is the vacuum dielectric constant, *ε* = 80 is the dielectric constant of water, *k* is the Debye constant which for a salt concentration *c* = 150 mM NaCl has a value at room temperature of *k* = 1.26 × 10^9^ m^−1^ and *σ*_A_ is the charge density in coulomb per square meter. Equation (12) is valid for very high ionic strength and low fractions of charged lipid (e.g., 10 %). The values for the charge density are different between the fluid and gel phases, and therefore, during the soliton propagation, there is a change in the electric potential. The area per lipid in the fluid and gel phases is respectively *A*_*f*_ = 0.629 and *A*_*g*_ = 0.474 nm^2^, and charged lipid has typically 1 or 2 electron charges (*e* = 1.602 × 10^−19^ C) [62]. If *N*_*T*_ is the total number of lipid in the membrane and 10 % of them are charged, then the charged density in the fluid phase is *σ*_A,fluid_ = −0.1 × e/ *A*_*f*_ = −0.025 C/m^2^ and in the gel phase is *σ*_A,gel_ = −0.1 × e/A_g_ = −0.034 C/m^2^. By substituting these values in Eq. (12), one obtains a potential change of about 10 mV. This is a very rough estimate since the exact number of charged lipid and their arrangement in the membrane are not known, the protein charges and dipole moments of the lipid molecules have not be considered.

The main weakness of the soliton model is that it cannot explain the role of the voltage-gated ion channels in the generation and propagation of the AP. During phase transition, lipid ion channels form spontaneously in the lipid membrane and they are very similar to protein ion channels [69]. Heimburg and collaborators have argued that the ionic currents observed during AP propagation might be related to these lipid ion channel currents also. However, this hypothesis remains to be demonstrated.

### Action potential: flexoelectricity hypothesis

Flexoelectricity is a concept similar to piezoelectricity. In flexoelectricity, the polarization charges across the membrane surface are induced by membrane bending. As for piezoelectricity, flexoelectricity exists as direct and reverse effect. In the direct effect, a change in the membrane curvature *d*C induces a change in the membrane potential proportional to *d*C as [70]

13 $$ {V}_{\mathrm{flexo}}=\frac{f_{{}_{f\mathrm{lexo}}}^D}{\varepsilon_0}d\mathbf{C} $$

with ${f}_{{}_{\mathrm{flexo}}}^D$ direct flexoelectric coefficient. The direct flexoelectric coefficient has been measured for some membranes. For example, the flexoelectric coefficient for the locust muscle membrane, which is an excitable membrane, was found to be *f*_flexo_ = 2.5 × 10^− 18^C [71]; about two orders of magnitude larger than the one for rat astrocyte membrane (non-excitable) having a coefficient *f*_flexo_ = 6.2 − 8.9 × 10^− 21^C [72]. The flexoelectric charge on the opposite side of the membrane capacitor taking also into account the change of the capacitance *C*_M_ with curvature is $q={C}_{\mathrm{M}}{V}_{\mathrm{flexo}}+\left(V+{V}_{\mathrm{flexo}}\right)\frac{\partial {C}_M}{\partial \mathbf{C}}d\mathbf{C}$ with *V*_flexo_ given by Eq. (13) and *V* applied voltage. The flexoelectric current is

14 $$ {I}_{\mathrm{flexo}}=\frac{d}{dt}q={C}_{\mathrm{M}}\frac{f_{\mathrm{flexo}}}{\varepsilon_0}\frac{d\mathbf{C}}{dt}+\left(V+{V}_{\mathrm{flexo}}\right)\frac{\partial {C}_M}{\partial \mathbf{C}}\frac{d\mathbf{C}}{dt} $$

In the reverse flexoelectric effect, the curvature C associated with the application of a transmembrane voltage *V* is [73]

15 $$ \mathrm{C}=\frac{f_{{}_{\mathrm{flexo}}}^R}{D{K}_B}V $$

with ${f}_{{}_{\mathrm{flexo}}}^R$ reverse flexoelectric coefficient, *K*_*B*_ bending modulus of membrane, and *D* membrane thickness. The reverse flexoelectric effect has been proved by Sachs and collaborators [74, 75] in whole-cell voltage-clamp experiments in human embryonic kidney cells using the atomic force microscope (AFM) for motion recording. From their data, it can be inferred a reverse flexoelectric coefficient of about ${f}_{{}_{\mathrm{flexo}}}^R\cong {10}^{-19}\mathrm{C}$ for this non-excitable membrane [76].

Petrov [76] has proposed the idea that the mechanical changes associated with the AP propagation might arise from the flexoelectrical property of the cell membrane. In his hypothesis, the voltage-gated ion channels still play a fundamental role in the generation and propagation of the AP and lipid phase transitions play no role. A strong enough membrane depolarization induces ionic currents through the voltage-gated ion channels, as in the H–H model, and at the same time induces a change of the membrane curvature through the inverse flexoelectric effect (Eq. 15). Therefore, he has argued that the AP is a flexoelectric wave, but to the best of our knowledge, no mathematical model has been developed to describe the propagation of this wave along the axon membrane.

The reverse flexoelectricity effect may provide a qualitative explanation of the observed increase in the nerve thickness and shortening of the nerve observed during AP propagation. Assuming the flexoelectricity coefficient of the axon membrane to be positive, then voltage change of about ΔV = 100 mV during AP propagation will induce a flexoelectric torque $t=-{f}_{{}_{\mathrm{flexo}}}^R\frac{\ \varDelta V}{D}$ that reduces the overall curvature by increasing its radius [76]. For a local segment of nerve membrane of radius *r*_0_ and length dx_0_, the nerve membrane curvature (for a cylindrical shape) is **C**_0_ = 1/*r*_0_*.* A decrease of the local curvature to **C** = 1/(*r*_0_ + dr), increase the radius to *r*_0_ + dr. If the volume of the nerve segment remains constant, *r*_0_^2^dx_0_ = *r*^2^dx, then dx must be less than dx_0_ and the nerve shortens locally.

In addition, changes in the local curvature of the nerve membrane can produce a change in the transmembrane potential through the direct flexoelectric effect (Eq. (13)) and induce the flexoelectric current given by Eq. (14). However, no equation has been developed yet able to predict the generation and propagation of the AP by changing of the local membrane axon curvature through the direct flexoelectric effect.

### Action potential: NICE model

The NICE model was proposed by Plaksin, Shoham, and Kimmel in 2014 [36]. This model modifies the H–H model to include the bilayer sonophore model [77] which describes as the response of the lipid membrane to ultrasound. The bilayer sonophore model predicts that ultrasound can induce expansions and contractions of the intramembrane space between the two leaflets of the lipid membrane resulting in membrane area changes proportional to the applied pressure amplitude and inversely proportional to the square root of frequency. This model predictions were experimentally supported using transmission electron microscopy of multilayered live-cell goldfish epidermis exposed in vivo. The expansion and contraction of the intramembrane space is modeled by the Rayleigh–Plesset equation for bubble dynamics and a diffusion equation determining the rate of transport of dissolved gas into and out of the lipid bilayer membrane. At sufficient high intensity levels (>100 mW/cm^2^) and frequencies where the cavitation effect dominates (about 1 MHz or less), ultrasound can induce intramembrane cavitation or nanobubble formation in the intramembrane space between the two lipid leaflets of cell’s membrane. The negative pressure of the ultrasound wave pulls the two monolayers of the lipid bilayer apart while the positive pressure pushes the monolayers towards each other; dissolved gas accumulates in the hydrophobic zone, creating nanobubbles that expand and contract periodically. The oscillations of these nanobubbles can induce changes in the local curvature of the membrane.

**Fig. 6:**
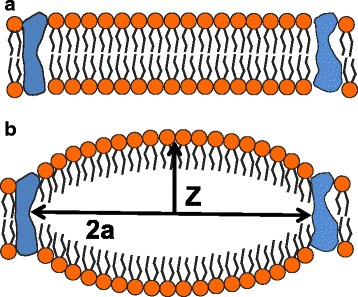
**a** Patch of lipid bilayer between two proteins. **b** Intramembrane cavitation induces change in the local curvature of the membrane

In the H–H model, the membrane capacitance *C*_M_ is constant, and therefore, the capacitive current ${I}_C=V\frac{d{C}_M}{dt}$ is zero. In the NICE model on the other hand, this capacitive current is different from zero and it is an alternating current (AC) flowing across the lipid membrane. This AC current is generated by the oscillations in the local curvature of the membrane induced by the intramembrane nanobubbles. According to the bilayer sonophore model, the membrane capacitance as a function of curvature can be expressed as

16 $$ {C}_{\mathrm{M}}\left(\mathbf{C}\right)=\frac{C_{\mathrm{M}}D}{a^2}\left[\mathbf{C}+\frac{a^2-\mathbf{C}-\mathbf{C}D}{2\;\mathbf{C}} ln\left(\frac{2\mathbf{C}+D}{D}\right)\right] $$

where C *=* C (*t*) is the membrane curvature shown in Fig. 6, *C*_M_ and *D* are respectively the capacitance and thickness of the membrane at rest, and *a* is the radius of a round patch of membrane. This current maybe thought as a flexoelectric current since it arises from the change of the local curvature of the membrane and it is the second term in the expression of the flexoelectricity current, Eq. (14), with *V*_flexo_ = 0.

The AP equation in the NICE model, consists of the H–H model equation, valid for cortical pyramidal neurons modified by the above capacitive AC current to hold:

17 $$ \frac{dV}{dt}=-\frac{1}{C_{\mathrm{M}}}\left[V\frac{d{C}_{\mathrm{M}}}{dt}+{g}_{\mathrm{Na}}\left(V-{E}_{\mathrm{Na}}\right)+{g}_{\mathrm{K}}\left(V-{E}_{\mathrm{K}}\right)+{g}_{\mathrm{M}}\left(V-{E}_{\mathrm{K}}\right)+{g}_{\mathrm{L}}\left(V-{E}_{\mathrm{L}}\right)\right] $$

where *g*_Na_, *g*_K_, *g*_M_, and *g*_L_ are the conductance of the sodium, delayed-rectifier potassium, slow non-inactivating potassium, and the leak channels, respectively, and the *E*_Na_, *E*_K_, and *E*_L_ are the equilibrium potentials.

**Fig. 7:**
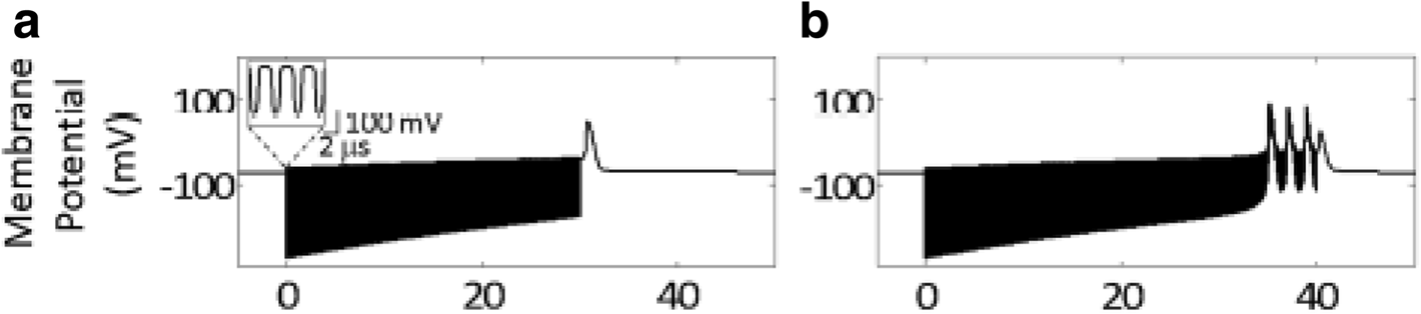
Solutions of the AP equation in the NICE model for a pulsed sinusoidal ultrasound wave with central frequency 0.35 MHz, pressure amplitude 100 kPa, intensity 320 mW/cm^2^, and pulse duration: **a** 30 ms, **b** 40 ms (After Plaksin et al. [36])

Equation (17) admits solutions describing generation of the AP by ultrasound. For example, a pulsed sinusoidal ultrasound wave with central frequency of 0.35 MHz, pressure amplitude of 100 kPa, intensity of 320 mW/cm^2^ and pulse duration of 30 ms, induces membrane hyperpolarization with very fast oscillations of the membrane potential. At the end of the ultrasound 30 ms pulse, membrane depolarization occurs that is sufficient to trigger the AP (Fig. 7a). For an ultrasound pulse of 40 ms, membrane depolarization occurs just before the end of the ultrasound pulse and the AP is generated (Fig. 7b).

## Discussion

In the following discussion, we will make a distinction between acoustic neuromodulation and acoustic neurostimulation, where acoustic neuromodulation is defined as a change of the electrical activity of neurons (e.g., modification of ionic currents and associated AP) under the influence of an acoustic stimulus and acoustic neurostimulation is defined as the occurrence of the electrical activity of neurons (e.g., initiation of AP) by the direct influence of an acoustic stimulus. There is experimental evidence that ultrasound can induce acoustic neuromodulation mostly in the form of suppression or reduction of electrical activity rather than in the form of its initiation. In the earlier animal studies, no neurostimulation of brain structures by FUS has been produced under sonication through the skull window. Recently, it has been reported that short pulses of low-intensity FUS can induce motor activity upon insonation of the cerebral cortex of mice [20–22] and rats [23] through the intact skull. Since, it is impossible to separate reliably the activities related to neurons and muscles, these results do not provide convincing evidence of FUS stimulation of brain structures [14], and results of control experiments with sonications through skull windows should be presented.

Here, we discuss the implications of the reviewed models and hypothesis described in our paper on FUS-induced neuromodulation and neurostimulation of AP. The soliton model is able to explain most of the thermodynamic findings on nerves associated with nerve signal propagation that cannot be explained with the H–H model. In the soliton model, the AP is a propagating density pulse (soliton), and it is an electromechanical rather than a purely electrical phenomenon. This thermodynamic model postulates that all the intensive thermodynamic variables can affect the state of the neuronal membrane. In addition, all these intensive thermodynamic variables change during AP propagation [78]. Any intensive thermodynamic variable that moves the membrane within transition should be able to generate AP. On the contrary, any intensive thermodynamic variable that pushes the membrane away from phase transition should inhibit AP. Ultrasound can induce thermal effect and mechanical effects, primarily through radiation force and cavitation. Depending on the exposure conditions, one effect can dominate over the others. In the context of the soliton model, acoustic neuromodulation may be considered a natural consequence of changes in temperature, pressure, or radiation pressure induced by ultrasound on the membrane. For example, if the local patch of axon is sonicated by FUS at exposure conditions where thermal effects are significant, then the lipids in membrane become more fluid. Therefore, membrane thickness decreases and area increases affecting the capacitive current and probably resulting in modulation of AP. These concepts may help in understanding the extensive studies performed by Lele [13] on more than 450 samples of peripheral nerves of cats, monkeys, earthworms, and men. He demonstrated that ultrasound exposure induced three phases of the action potential (AP) changes that could be duplicated by heating of nerve segment: reversible enhancement (T <41 °C), reversible suppression (41 °C < T < 45 °C), and irreversible suppression at temperatures which were high enough to block the AP completely. Furthermore, due to increased thermal fluctuations, the probability of pore occurrence in the lipid membrane increases as a function of temperature; also affecting capacitive currents and therefore the AP. Sonoporation, which has been investigated primarily for enhancing drug delivery, can also affect membrane potential and induce ionic currents across the membrane as was shown, for example, by Deng et al. [79] in an in vitro study on Xenopus oocyte. To the best of our knowledge, sonoporation has never been specifically investigated for neuromodulation.

For neurostimulation (generation of the AP) to occur in the soliton model, a local patch of axon membrane has to be brought within phase transition. The application of an adequate acoustic pressure that decreases the area of a local patch of membrane and increases its thickness can bring the membrane within the phase tradition, thus initiating the AP. The effects of FUS on lipid membrane phase transition need to be demonstrated and exposure conditions enable to exert adequate acoustic pressure has to be investigated.

Furthermore, we suggest that axon membranes can be considered as a form of liquid crystals, which have the ability to generate an electric charge in response to applied mechanical stress. In piezoelectrics, voltage changes and density changes are tightly coupled. Such coupling between lateral density and electric potential is known as electromechanical coupling, it is linked to changes in capacitance. If the axon membrane has piezoelectric properties, changes of membrane area density induce voltage changes, which are proportional to membrane area density changes, and the soliton pulse, according to [64], will propagate as a piezoelectric wave. However, we found no valid evidence supporting this suggestion; therefore, this problem is unsolved and needs to be investigated theoretically and experimentally.

Electromechanical coupling in membranes was first proposed by Petrov [35, 70]. The direct and reverse flexoelectric effects have been both proved experimentally to occur in artificial lipid membranes and in cells membranes (for a review, see [76]). However, how flexoelectricity affects AP generation and propagation needs to be investigated. In direct flexoelectricity, changes in the local radius of curvature of the nerve can influence the transmembrane potential and induce a flexoelectric current. Therefore, it may be expected that changes in the nerve local radius of curvature induced by ultrasonic radiation force or cavitation can induce neuromodulation, both in the form of suppression or enhancement, since it will affect the transmembrane potential and induce flexoelectric currents. The relationship between the flexoelectric current and currents through voltage-gated ion channels is currently unknown. However, an amplification of the flexoelectric current of about 50 times was observed in a patch of locust muscle membrane containing K^+^ ion channels during channel opening [80].

Since a flexoelectric model of AP generation by direct flexoelectricity has not been developed yet, how cavitation or radiation force could induce neurostimulation or reversible suppression of the AP according to the flexoelectricity hypothesis is not known. Temperature does not play a role in the flexoelectricity hypothesis; therefore, neurostimulation and neuromodulation of the AP induced by ultrasound thermal effects cannot be discussed in this model.

The NICE model modifies the H–H model to include a capacitive current, ${I}_{\mathrm{C}}=V\frac{d{C}_{\mathrm{M}}}{dt}$, arising from the local change in membrane capacitance. This type of current has been observed experimentally. For instance, Ochs and Burton [81] showed that by applying an oscillating pressure across the lipid bilayer under voltage-clamp conditions, an alternating capacitive current was induced across the membrane. They interpreted this current as a result of the change in membrane area. Petrov and collaborators [67] instead interpreted this current as a flexoelectric current induced by the applied oscillating pressure. In their study, they considered only the first term in the expression of the flexoelectric current given by Eq. (14). In the NICE model, on the other hand, the second term in Eq. (14) is considered with *V*_flexo_ = 0. Therefore, in the NICE model, it is assumed that the membrane curvature modulation generated by intramembrane cavitation does not induce change in the flexoelectric potential but only changes in the membrane capacitance. The modulation of the membrane capacitance, initiated by intramembrane cavitation, can induce rapid AC hyperpolarizing currents that can lead to AP generation. Within the NICE model, cortical suppression, using low-duty cycle stimulation, is mediated through cell-type selective interactions [82]. The NICE model can explain [36] the data of an in vivo study by King et al. [21] where a wide range of US pulses were used to stimulate mouse primary motor cortex.

## Conclusions

We have discussed the soliton model, the flexoelectricity hypothesis, and the NICE model to provide an understanding of the non-electric aspects associated with generation and propagation of the AP. These models and hypothesis can offer insights on how a non-electric stimulus such as an acoustic stimulus might influence AP. In the soliton model, the AP is a sound soliton, propagating as piezoelectric wave, and all the intensive thermodynamic variables (temperature, voltage, pressure, shear pressure, and chemical potential) can in principle initiate or suppress the AP provided that they move a local patch of membrane within or out of the phase transition range. In the flexoelectricity hypothesis, a mechanical stimulus enables to modulate the curvature of a local patch of membrane can influence the transmembrane potential and modulate the AP. The NICE model suggests that the alternating current generated by oscillations in membrane curvature caused by intramembrane cavitation can initiate the AP.

Despite many unsolved problems, these models and hypothesis are very attractive and hopefully they will be further developed, because they provide matrices of assumptions by which further progress can be made in making sense of all diverse data.

## Ethics approval and consent to participate

Not applicable.

### Consent for publication personal data

Not applicable.

### Availability of data and supporting materials section

Not applicable.

## Abbreviations

AP: action potential
DPPC: dipalmitoylphosphatidylcholine
FUS: focused ultrasound
H–H model: Hodgkin–Huxley model
NICE model: neuronal intramembrane cavitation excitation model
SD: spreading depolarization
